# A new colorimetric lactate biosensor based on CUPRAC reagent using binary enzyme (lactate-pyruvate oxidases)-immobilized silanized magnetite nanoparticles

**DOI:** 10.1007/s00604-024-06531-w

**Published:** 2024-07-09

**Authors:** Selen Ayaz, Teslime Erşan, Yusuf Dilgin, Reşat Apak

**Affiliations:** 1 Department of Chemistry, Faculty of Science, Çanakkale Onsekiz Mart University, Canakkale, 17020 Turkey; 2 Department of Chemistry, Faculty of Engineering, İstanbul University-Cerrahpaşa, Avcılar, 34320 Istanbul, Turkey; 3 Turkish Academy of Sciences (TUBA), Bayraktar Neighborhood, Vedat Dalokay St. No: 112, Çankaya, 06690 Ankara, Turkey

**Keywords:** Colorimetric biosensor, CUPRAC reagent, Bienyzmatic biosensor, Magnetite nanoparticle, Lactate biosensors

## Abstract

A novel optical lactate biosensor is presented that utilizes a colorimetric interaction between H_2_O_2_ liberated by a binary enzymatic reaction and bis(neocuproine)copper(II) complex ([Cu(Nc)_2_]^2+^) known as CUPRAC (cupric reducing antioxidant capacity) reagent. In the first step, lactate oxidase (LOx) and pyruvate oxidase (POx) were separately immobilized on silanized magnetite nanoparticles (SiO_2_@Fe_3_O_4_ NPs), and thus, 2 mol of H_2_O_2_ was released per 1 mol of the substrate due to a sequential enzymatic reaction of the mixture of LOx-SiO_2_@Fe_3_O_4_ and POx-SiO_2_@Fe_3_O_4_ NPs with lactate and pyruvate, respectively. In the second step, the absorbance at 450 nm of the yellow-orange [Cu(Nc)_2_]^+^ complex formed through the color reaction of enzymatically produced H_2_O_2_ with [Cu(Nc)_2_]^2+^ was recorded. The results indicate that the developed colorimetric binary enzymatic biosensor exhibits a broad linear range of response between 0.5 and 50.0 µM for lactate under optimal conditions with a detection limit of 0.17 µM. The fabricated biosensor did not respond to other saccharides, while the positive interferences of certain reducing compounds such as dopamine, ascorbic acid, and uric acid were minimized through their oxidative removal with a pre-oxidant (NaBiO_3_) before enzymatic and colorimetric reactions. The fabricated optical biosensor was applied to various samples such as artificial blood, artificial/real sweat, and cow milk. The high recovery values (close to 100%) achieved for lactate-spiked samples indicate an acceptable accuracy of this colorimetric biosensor in the determination of lactate in real samples. Due to the increase in H_2_O_2_ production with the bienzymatic lactate sensor, the proposed method displays double-fold sensitivity relative to monoenzymatic biosensors and involves a neat color reaction with cupric-neocuproine having a clear stoichiometry as opposed to the rather indefinite stoichiometry of analogous redox dye methods.

**Graphical Abstract:**

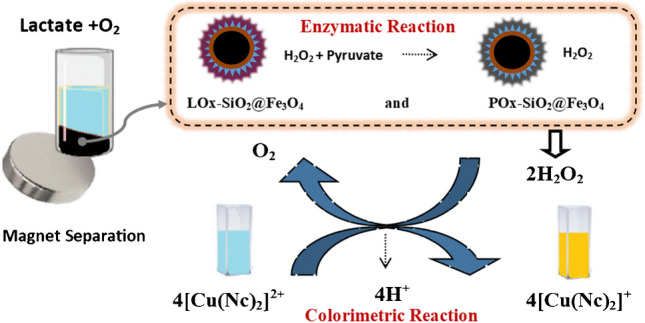

**Supplementary Information:**

The online version contains supplementary material available at 10.1007/s00604-024-06531-w.

## Introduction

A biosensor is a device that combines a transducer with a bioreceptor to assess the concentration or activity of the analyte in the sample. In biosensors, the signal received from the transducer is reflective of the specificity of the bioreceptor and proportional to the concentration of the target analyte [1, 2]. Among the biosensors, more studies have been carried out on those whose receptors are enzymes [3]. Enzymes are the best catalysts used as biologically active materials due to their high selectivity and substrate-directed activity; many biochemical reactions are catalyzed by specific enzymes. Thus, enzymes are extensively used as catalytic components in biosensors [2, 4, 5]. Multienzymatic biosensors use two or more enzymes to perform a cascade of reactions to detect analytes. They have advantages over single enzyme-based biosensors regarding sensitivity, selectivity, and range of applications [6]. The limited use of enzymes in biosensors is due to their need for more stability in environments of variable pH, temperature, and ionic strength [7]. Therefore, when designing enzyme-based biosensors, efforts are made to create an appropriate environment to sustain enzyme activity. In this context, various types of nanomaterials are used as carrier support in enzyme immobilization. Recently, magnetic Fe_3_O_4_ nanoparticles have found great interest in enzyme immobilization due to their chemical, physical, thermal, magnetic, and mechanical properties, as well as the convenience and improved properties they offer to various processes [8, 9]. Some benefits of utilizing the surface of magnetic Fe_3_O_4_ nanoparticles for enzyme immobilization are as follows: (i) improved stability and durability: immobilization of enzymes on magnetic nanoparticles yields more stable and durable structures than their free form [10, 11]. The magnetic nanoparticles protect the enzymes from harsh environments such as pH changes, organic solvents, and high temperatures [11]. (ii) Ease of separation: facilitates recovery and re-use of immobilized enzymes in reactions [12]. Enzyme-immobilized magnetite nanoparticles can easily be separated from the solution environment by a magnet after the enzymatic reaction is completed. This makes them a cost-effective and environmentally friendly alternative to traditional enzyme immobilization methods. (iii) Improvement of the catalytic activity: enzymes immobilized on magnetic nanoparticles show an improved catalytic activity compared to their free form [13, 14]. (iv) Recyclability: magnetic nanoparticles can be reused multiple times without significant activity loss. Due to these advantages, the immobilization of enzymes on the surface of magnetic nanoparticles is of great interest, and Fe_3_O_4_ nanoparticles are often integrated into enzymatic biosensors [11–13].

Lactate is a crucial metabolite formed from the anaerobic metabolism of glucose in muscles [15]. The production of lactic acid leads to an elevation in the concentration of protons within cells. If the rate of lactic acid production is sufficiently high, it may surpass the cellular capacity to buffer protons, causing a decrease in cellular pH. This can result in cell acidosis, which negatively affects muscle function [16]. Therefore, monitoring lactate levels can help athletes optimize their training and evaluate their performance endurance, the so-called lactate threshold [16, 17]. Lactate biosensors are devices that detect lactate levels in biological fluids. Lactate levels in intensive care units and operating rooms can serve as a diagnostic indicator for patient problems [16, 18]. Due to such applications, the significance of lactate biosensor studies has increased in recent years. To quantify lactate in a wide variety of samples and matrices, numerous analytical methods have been developed by using chromatographic [19, 20], electrochemical [21–23], and optical techniques such as spectrophotometry [24], colorimetry [25–27], fluorimetry [28], and chemiluminescence [29]. Recently, colorimetric biosensors have been preferred over other analytical techniques due to their ease of fabrication, rapid detection, high sensitivity, and selectivity, as well as their ability to permit naked-eye sensing [30, 31], especially in point-of-care testing applications. This type of biosensor is based on the change of color in a reaction mixture due to the formation of a colored product. They are simple to use and do not require any expensive equipment [30] as well as being user-friendly and providing rapid results [31]. In this context, enzyme-based colorimetric biosensors have found great interest in the accurate, selective, and sensitive determination of lactate due to the above-mentioned advantages of enzymes as biorecognition elements [31].

The cupric reducing antioxidant capacity (CUPRAC) reagent was invented by the Apak research group in 2004 to measure the total antioxidant capacity (TAC) of phenolic compounds [32]. It is a bis(neocuproine)copper(II) chelate ([Cu(Nc)_2_]^2+^) and has been extensively used as a chromogenic oxidizing agent in the development of a colorimetric and spectrophotometric sensor for the determination of various compounds. At the end of the colorimetric reaction between the CUPRAC reagent and reductant analyte, [Cu(Nc)_2_]^2+^ is reduced to yellow-orange-colored bis(neocuproine)copper(I) chelate ([Cu(Nc)_2_]^+^), while analyte is oxidized. Thus, the colorimetric or optical sensors have been developed based on the measurement of absorbance of [Cu(Nc)_2_]^+^ at 450 nm, where the complex gives maximum absorbance.

In our recent studies, the colorimetric CUPRAC reaction and enzymatic reactions based on various types of enzymes were integrated for the first time, and highly sensitive and selective colorimetric biosensors based on this system have been developed. For example, glucose, uricase, and xanthine biosensors based on oxidases [33, 34], glucose biosensors based on glucose dehydrogenase [35], and enzymatic organophosphate pesticide biosensors based on the inhibition activity of pesticide toward acetylcholine esterase [36] have been reported. However, our literature search shows that no such study has been carried out with lactate oxidase (LOx) and pyruvate oxidase (POx) enzymes for the fabrication of lactate biosensors. Although many electrochemical lactate biosensors based on the use of LOx [37–39], lactate dehydrogenase (LDH) [40–42], and multi-enzymes such as LDH/POx [23] and LOx/POx [43] have been constructed, there are only limited studies on bienzymatic biosensors for the optical or colorimetric determination of lactate. Although the bienzymatic mode of study may provide synergistic enhancement in sensitivity and selectivity of analysis, it is also a challenge requiring extra care for the optimization of analytical protocols.

In this proposed work, a new optical lactate biosensor has been developed based on a colorimetric reaction between H_2_O_2_ generated by two enzymes (LOx and POx) and [Cu(Nc)_2_]^2+^ for the first time. The successful combination of two enzymes on a magnetic nanoparticle, creating a new nanoprobe that allows selective and sensitive lactate determination, demonstrates the novelty of this research. This newly developed nanoprobe shows excellent activity in the colorimetric interaction between the CUPRAC reagent and H_2_O_2_ released by a binary enzymatic reaction of double-fold sensitivity. Furthermore, Cu(II)-neocuproine reacts with H_2_O_2_ without activation, i.e., H_2_O_2_ does not require to be catalytically converted by a peroxidase into reactive oxygen species (ROS) to eventually colorize redox dyes such as tetramethylbenzidine (TMB) which would create several species in equilibrium that are responsible for simultaneous light absorption. On the other hand, most recent colorimetric biosensors for lactate determination utilize the lactate/lactate oxidase reaction producing H_2_O_2_ activated by peroxidase and eventually detected with TMB [44, 45], but this peroxidase/TMB system gives rise to certain difficulties. Firstly, Josephy et al. established that at less than equimolar peroxide, all four species derived from TMB (diamine, radical cation, charge-transfer complex, and diimine) exist in equilibrium [46] which means that light absorption in the visible range will be the sum of the absorbances of several species having different molar absorptivities at the selected analytical wavelength, resulting in inevitable deviations from Beer’s law and partial loss of linear response of absorbance vs. concentration [44, 45]. Secondly, H_2_O_2_ needs to be activated by peroxidases or mimics into ROS in order to colorize TMB, and peroxidase activators are easily inhibited with thiols [47], flavonoids [48], and several metal ions. It is worthy of notice that the proposed methodology does not suffer from any of the outlined problems and determines the H_2_O_2_ produced (from enzyme–substrate reaction) with a net CUPRAC reaction of definite stoichiometry and excellent linearity free from interferences to Beer’s law. These unique properties highlight its potential for biosensor applications, especially lactate detection. Accurate, sensitive, fast, simple, and more economical lactate determination will pave the way for more accurate and timely monitoring of athletes, contribute to the diagnosis and treatment of related diseases in a short time by monitoring general patient results, and improve health services. All these aspects reflect the importance of this study and its necessity in the fields of health, sports, and even food. Bienzymes (LOx and POx) catalyzed the oxidation of lactate and then pyruvate resulting in the formation of H_2_O_2_. This biosensor measures lactate concentration by monitoring the change in absorbance of the product (reduced form of Cu(II) complex, i.e., [Cu(Nc)_2_]^+^) of the colorimetric reaction that takes place between H_2_O_2_ generated by the enzymatic reactions and the CUPRAC reagent. In the proposed methodology, the CUPRAC reagent acts as a neat 2e^−^ oxidant toward H_2_O_2_ to stoichiometrically produce oxygen (O_2_) without side reactions and give rise to a single light-absorbing product, i.e., cuprous-neocuproine, as opposed to the indefinite stoichiometry of redox dyes that produce colored products from peroxidase (or mimic)-catalyzed degradation of H_2_O_2_.

## Materials and methods

### Reagents and apparatus

Lactate oxidase from *Aerococcus viridans* (LOx, lyophilized powder, 45 U/mg solid), pyruvate oxidase from microorganisms (POx, lyophilized powder, 7.4 U/mg solid), 2,9-dimethyl-1,10-phenanthroline (neocuproine), sodium L-lactate, NH_4_CH_3_COO, and CuCl_2_ were purchased from Sigma-Aldrich. The other reagents used were of analytical grade. An enzyme solution is composed of 1.0 mg/mL of LOx and 2.0 mg/mL POx in 1.0 mL of 1.0 M NH_4_CH_3_COO buffer (pH 7.0). A stock solution of sodium lactate (5.0 mM) was prepared by weighing out the right amounts and diluting them in water until they reached a known volume. To record spectra and to measure absorbance, a Shimadzu UV-1208 UV–VIS spectrophotometer and an Ocean Insight HR4Pro spectrophotometer with an Ocean Insight DH-2000-BAL UV–VIS-NIR light source were used. An Elga Option Q7B water purification system obtained deionized water with a resistivity of 18.2 MΩ.cm.

### Fe_3_O_4_ nanoparticle synthesis and enzyme immobilization

Fe_3_O_4_ nanoparticles were prepared using an established procedure reported in our previous study [35, 49]. Briefly, FeSO_4_·7H_2_O and FeCl_3_·6H_2_O were dissolved in HCl, and then, aqueous ammonia was added under shaking of the solution under the Ar atmosphere. The black-colored Fe_3_O_4_ was separated, washed, and dried. For silanization, Fe_3_O_4_ was coated with tetraethyl orthosilicate (TEOS) and 3-aminopropyl triethoxysilane (APTES), then magnetically separated, washed, and dried [35, 50]. The resulting SiO_2_@Fe_3_O_4_ nanoparticles were then stored in a vacuum at room temperature [35]. The enzyme immobilization process involved adding 15 mg SiO_2_@Fe_3_O_4_ NPs to a 1% (w/v) chitosan solution, stirring, and washing. The nanoparticles were then treated with glutaraldehyde (GAL) and an optimized amount of enzymes. The enzyme-immobilized nanoparticles were prepared for use by washing with BSA, magnet separation, and pH 5.0 PBS [35]. Detailed surface characterization of silanized magnetite nanoparticles using SEM, TEM, EDX, XRD, and XPS has already been reported in our previous study [35].

### Spectrophotometric measurements

In the first step, spectrophotometric measurements relied on the colorimetric reaction between [Cu(Nc)_2_]^2+^ and pure H_2_O_2_ which was not produced from an enzymatic reaction. The absorbance of the yellow-colored [Cu(Nc)_2_]^+^ complex generated as a consequence of the reaction was measured at a wavelength of 450 nm. Then, enzymatic biosensor studies were performed by using LOx/POx, and enzymatic reaction was let to take place in the solution environment (enzymes were added into solution media without using an immobilization process). In this second step, some parameters such as pH, concentrations of CuCl_2_, and neocuproine for the colorimetric reaction and pH, temperature, amount of enzymes (LOx and also POx), and reaction time for the enzymatic reaction were optimized.

In the third step, lactate biosensor studies were performed using enzyme-immobilized magnetite nanoparticles. Thus, spectrophotometric measurements were conducted to investigate the color reaction between the CUPRAC reagent and the H_2_O_2_, the latter being generated by the enzymatic reaction of the monoenzyme (LOx)- and also bienzyme-immobilized magnetite nanoparticles (LOx-SiO_2_@Fe_3_O_4_ and POx-SiO_2_@Fe_3_O_4_ NPs) in the presence of substrate (lactate). For this purpose, the amount of enzymes to be immobilized, the amount of Fe_3_O_4_, and the enzymatic reaction time were optimized, and analytical performance studies were carried out for each enzyme-immobilized magnetite nanoparticle. In this context, various volumes of 5.0 mM lactate solution were added in 0.75 mL of 1.0 M NH_4_CH_3_COO, including 15.0 mg LOx-SiO_2_@Fe_3_O_4_ NPs or the mixture LOx-SiO_2_@Fe_3_O_4_ and POx-SiO_2_@Fe_3_O_4_ nanomaterials (15 mg each). After the enzymatic reaction was completed, the enzyme-immobilized magnetite nanoparticles were separated with a magnet. Then, the CUPRAC reagent (0.75 mL of 2.25 mM Nc dissolved in ethanol and 0.50 mL of 2.0 mM CuCl_2_) was added to the separated solution for the colorimetric reaction to occur. After the final solution volume was completed to 2.5 mL with water, the lactate biosensor was established based on measuring the absorbance at 450 nm of the yellow-orange [Cu(Nc)_2_]^+^ complex formed in the colorimetric reaction.

### Real sample analysis

The designed lactate biosensor was used with a variety of samples, such as artificial/real sweat, cow milk, and artificial blood. The samples of artificial sweat [51] and artificial blood [35] were re-prepared in a 50-mL volumetric flask according to the literature to contain 1.5 mM sodium lactate. The real sweat sample was obtained in a 2 mL volume from the author (Selen Ayaz), who perspired sufficiently after walking at a high speed for a minimum of 30 min [26]. The cow milk sample (200 mL) was purchased from a commercial supermarket. In the lactate biosensor, the amount of lactate in cow milk, artificial blood, and artificial/real sweat samples was determined by measuring the absorbance values at 450 nm after enzymatic and colorimetric reactions using bienzyme-immobilized, silanized magnetite nanoparticles (LOx-SiO_2_@Fe_3_O_4_ + POx-SiO_2_@Fe_3_O_4_).

## Results and discussion

### Spectrophotometric assay based on the colorimetric reaction between enzymatically produced H_2_O_2_ and [Cu(Nc)_2_]^2+^

To investigate the colorimetric reaction between enzymatically produced H_2_O_2_ and [Cu(Nc)_2_]^2+^, firstly, certain parameters such as amounts of enzymes, pH, temperature, and enzymatic and colorimetric reaction times were optimized in the use of the enzymes in solution media (i.e., without immobilization of enzyme). In the colorimetric reactions, a previously optimized procedure (750 μL of 2.25 mM Nc prepared in ethanol + 500 µL of 2.0 mM CuCl_2_ prepared in pure water, 750 μL of 1.0 M NH_4_CH_3_COO at pH 7.0, *x* μL of solution obtained at the end of the enzymatic reaction and (500 − *x*) μL of water) was used. The absorbance of the yellow-orange [Cu(Nc)_2_]^+^ complex formed after the indirect reaction of [Cu(Nc)_2_]^2+^ with 50 µM lactate (i.e., after conversion with oxidase enzymes) was spectrophotometrically measured. The curves obtained from optimization studies showed that maximum absorbance values were obtained in the use of 25 µL of 1.1 mg/mL LOx, 50 µL of 2.0 mg/mL POx, pH 6.0 or 7.0, 1.0 M NH_4_CH_3_COO, 25 °C temperature, 45 min enzymatic reaction time, and 1 min colorimetric reaction time (Fig. S1). Under these optimized conditions, analytical performance studies were carried out for pure H_2_O_2_ (without production from enzymatic reaction) and for lactate using LOx in which H_2_O_2_ was released proportionally with increasing concentrations of lactate at the end of enzymatic reaction (Figs. S2 and S3). The results obtained from absorbance measured at 450 nm show that the linear calibration plots were found in the range from 1.0 to 125.0 μM for H_2_O_2_ with an equation of *A* = 0.0126 C (μM) + 0.0058 (Fig. S2), in the range from 1.0 to 100.0 μM for lactate using LOx with an equation of *A* = 0.0107 C (μM) ± 0.0019 (Fig. S3). The slopes of the lines obtained for H_2_O_2_ formed as a result of the enzymatic reaction of lactate and pure H_2_O_2_ were relatively close to each other. This result supports the fact that in the presence of LOx, H_2_O_2_ was formed proportionally depending on lactate concentration, and the resulting H_2_O_2_ reacted with [Cu(Nc)_2_]^2+^ to form yellow-colored [Cu(Nc)_2_]^+^. To determine the LOD and LOQ values, ten different 1.0 µM H_2_O_2_ and 1.0 µM lactate solutions were prepared, and the standard deviations of the absorbance values were calculated for each analyte. These standard deviations were accepted as the standard deviation of blank (*s*_blank_). The LOD and LOQ values for H_2_O_2_ were calculated as 0.37 µM and 1.24 µM, respectively, whereas the same values for lactate were found as 0.35 µM and 1.16 µM, respectively, using the following equations:

$$LOD = 3{s}_{blank}/slope$$

$$LOQ = 10{s}_{blank}/slope$$

### Studies on optical lactate biosensors based on LOx- and POx-immobilized SiO_2_@Fe_3_O_4_ nanoparticles

The studies continued with the synthesis [35, 49], silanization [35, 50], and enzyme immobilization [35] of magnetic nanoparticles according to the previously reported procedures. The LOx and POx enzymes were separately immobilized on magnetic nanoparticles which were previously silanized with TEOS + APTES (SiO_2_@Fe_3_O_4_), by using chitosan and then glutaraldehyde cross-linking procedures. Similar steps such as magnetite nanoparticle synthesis, silanization, and their detailed characterization were reported in our previous study, in which GDH was immobilized onto SiO_2_@Fe_3_O_4_ NPs [35]. In this study, monoenzyme (LOx)- and bienzyme (LOx + POx)-immobilized SiO_2_@Fe_3_O_4_ NPs were also characterized by FTIR, SEM, and EDX mapping (Figs. S4-S8). Then, the amount of LOx, the amount of SiO_2_@Fe_3_O_4_ nanoparticles, the enzymatic and colorimetric reaction times, pH, and temperature were optimized for monoezyme (LOx)-immobilized Fe_3_O_4_ NP-based biosensor. Absorbances of yellow-orange-colored [Cu(Nc)_2_]^+^ formed after the colorimetric reaction of enzymatically produced H_2_O_2_ (with the use of LOx only) with [Cu(Nc)_2_]^2+^ were measured at 450 nm. As shown in Fig. S9, maximum absorbance values for two different concentrations of lactate (25 and 50 µM) were found in the use of 0.3 mg of LOx, 15 mg of SiO_2_@Fe_3_O_4_, 15 min enzymatic reaction time, 1 min colorimetric reaction time, pH 6.0 or 7.0, and 25 °C temperature.

Magnetite nanoparticles (containing trace Fe(II)) themselves may reduce the Cu(II)-neocuproine complex to Cu(I)-neocuproine complex in the absence of substrate or H_2_O_2_ in the environment, changing it from pale blue to the yellow-orange color. To prevent this, H_2_O_2_ was produced by reacting the substrate with the bienzyme immobilized on magnetite nanoparticles. Then, the magnetite nanoparticles were separated from the solution with a magnet, and the colorimetric reaction was carried out by adding the CUPRAC reagent. In short, while the enzymatic reaction took place in the presence of magnetite nanoparticles resulting in the formation of H_2_O_2_, Fe_3_O_4_ NPs were absent during the colorimetric reaction between CUPRAC and enzymatically produced H_2_O_2_. In this case, any interference from the peroxidase-like properties of Fe_3_O_4_ NPs has been eliminated. In other words, magnetite nanoparticles were the enzyme carriers when needed (i.e., in the enzymatic reaction) and removed when not needed (i.e., in the colorimetric reaction).

As shown in Figs. S1C and E, respectively, the enzymatic reaction reaches equilibrium in about 40–45 min, while the colorimetric reaction between enzymatically produced H_2_O_2_ and the CUPRAC reagent takes place in about 1 min. This is due to the fact that the enzymatic reaction involves first the formation of a complex (intermediate product) between the enzyme and the substrate (ES) and then the transformation of this complex into the product (*E* + *P*), which is carried out by two consecutive enzymes (first, between LOx and lactate, and second, between enzymatically produced pyruvate and POx). Therefore, these complex equilibria are expected to take a longer time where new bonds are formed while old bonds are broken. However, in the colorimetric reaction, a rapid redox reaction with a very high equilibrium constant occurred directly between the CUPRAC reagent and H_2_O_2_ without the formation of any enzyme–substrate complex (intermediate products). On the other hand, when the enzymes were immobilized on magnetite nanoparticles, the enzymatic reaction time decreased to 10–15 min (Figs. S9 C and D). This is due to the improvement of the catalytic activity of enzymes by their immobilization on magnetic nanoparticles compared to their free form (e.g., a local enrichment in the concentration of the enzyme reagent immobilized on the nanoparticle surface increases the rate of the reaction), as previously described in the “Introduction” section [13, 14]. It can be seen in Fig. S1D that the absorbance value decreases slightly by increasing pH from neutral to 8.0 due to the decreasing activities of both LOx and POx in the basic environment. Similar results were obtained for LOx-based colorimetric and fluorimetric lactate biosensors [52, 53] and pyruvate oxidase-based phosphate biosensors [54]. In these studies, it was reported that the best enzyme activity was obtained between pH 6 and 7, and a decrease in signals was obtained due to the decrease in activity toward pH 8 and 9, which supports our results. It may be useful to mention that although the redox half-cell reaction between Cu(Nc)_2_^2+^ and Cu(Nc)_2_^+^ does not involve protons, the CUPRAC reaction best operates in a neutral solution, since increased basicity may cause partial hydrolysis of the less stable cupric complex, slightly decreasing the standard potential of the Cu(Nc)_2_^2+^/Cu(Nc)_2_^+^ redox couple with a subsequent decrease in the oxidizing power of cupric-neocuproine toward H_2_O_2_.

**Fig. 1:**
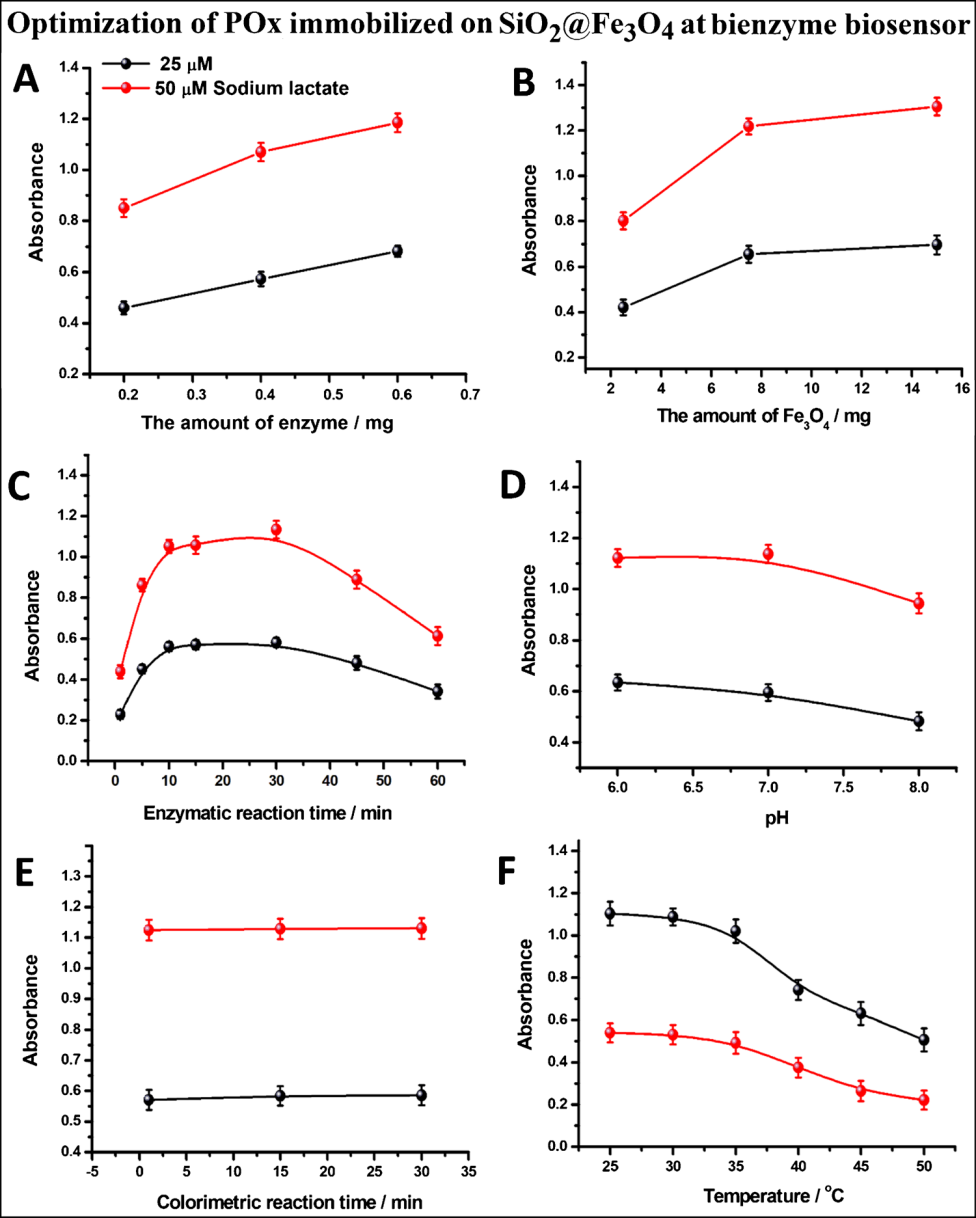
Optimization curves of **A** amount of enzyme immobilized POx on 15 mg SiO_2_@Fe_3_O_4_ and **B** amount of Fe_3_O_4_ using 0.6 mg POx in the presence of 15 mg LOx(0.3 mg)-SiO_2_@Fe_3_O_4_ NPs and following optimization curves of **C** enzymatic reaction time, **D** colorimetric reaction time, **E** pH, and **F** temperature using mixture of 15 mg LOx(0.3 mg)-SiO_2_@Fe_3_O_4_ NPs and POx(0.6 mg)-SiO_2_@Fe_3_O_4_ NPs

As seen from the experiments performed by using enzyme in solution media (i.e., without immobilization), when POx enzyme was used twice as much as LOx enzyme, the absorbance doubled as expected. This was supported by optimizing 0.6 mg of POx immobilized on 15 mg of SiO_2_@Fe_3_O_4_ used together with 0.3 mg LOx-immobilized SiO_2_@Fe_3_O_4_ (15 mg) for bienzyme-based biosensor (Fig. 1A). Then, the mixture of POx-SiO_2_@Fe_3_O_4_ and LOx-SiO_2_@Fe_3_O_4_ nanomaterials (each 15 mg) was used for the optimization of the enzymatic and colorimetric reaction times, pH, and temperature for bienzyme (LOx + POx)-immobilized Fe_3_O_4_ NP-based biosensor. As seen in Fig. 1B–E, a reaction time of 10–15 min, 1 min colorimetric reaction, pH 6.0 or 7.0, and 25 °C temperature were also found optimal parameters for the bienzyme-based lactate biosensor as in the case of monoenzyme. In addition, absorbance values increased twice compared to those obtained with monoenzyme due to the formation of 2 mol H_2_O_2_/1 mol substrate in the bienzymatic reaction, resulting in an increased amount of yellow-orange complexes with the chromogenic reagent, as expected. These results confirmed the enhanced analytical sensitivity achieved with bienzyme usage and also the stoichiometric character of both enzymatic and colorimetric reactions.

**Fig. 2:**
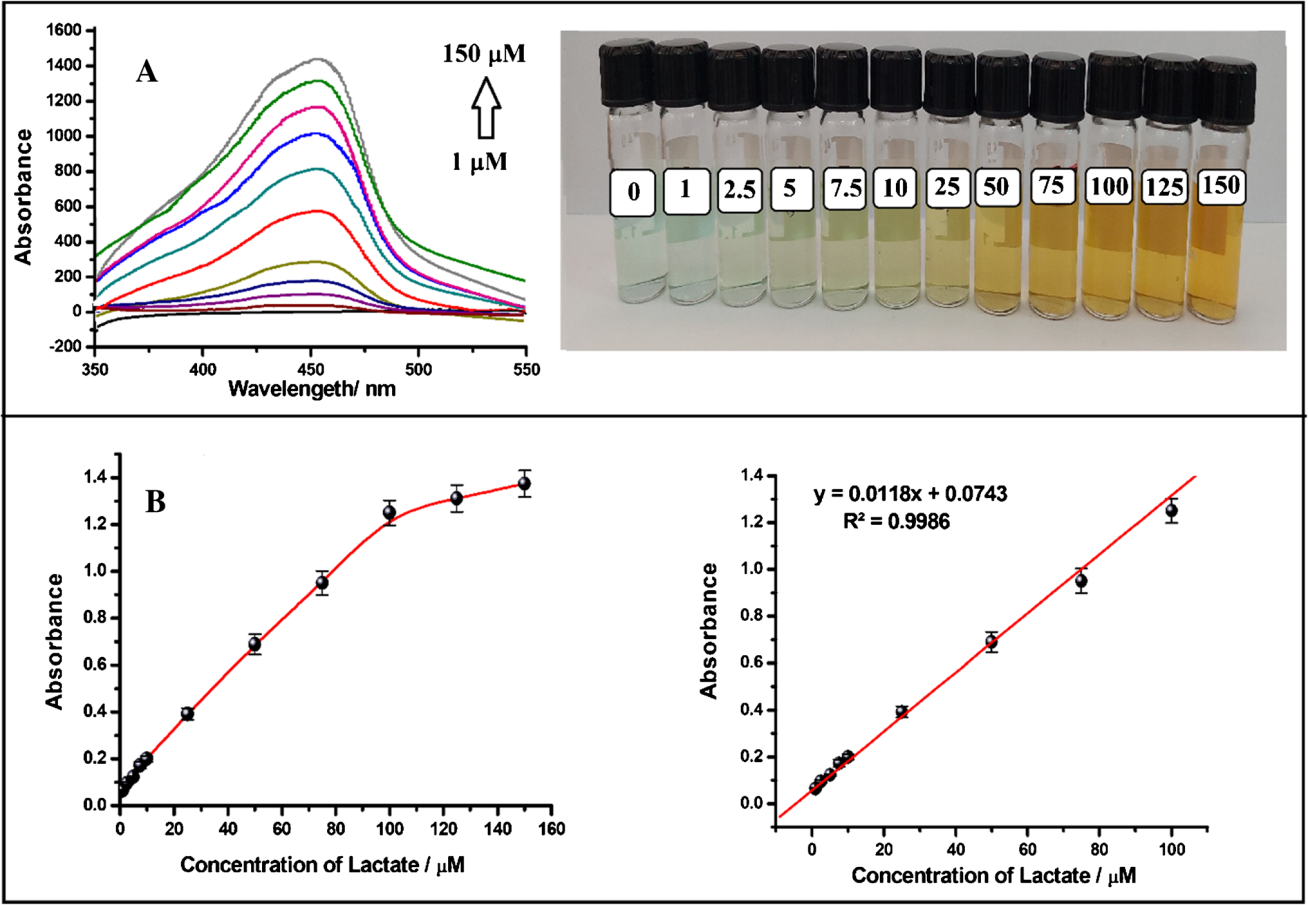
**A** Spectra and photographs recorded for lactate biosensor with increasing lactate concentration using monoenzyme-immobilized nanoparticles (LOx-SiO_2_@Fe_3_O_4_). **B** The curve of absorbance recorded at 450 nm versus lactate concentration and linear calibration plot

**Fig. 3:**
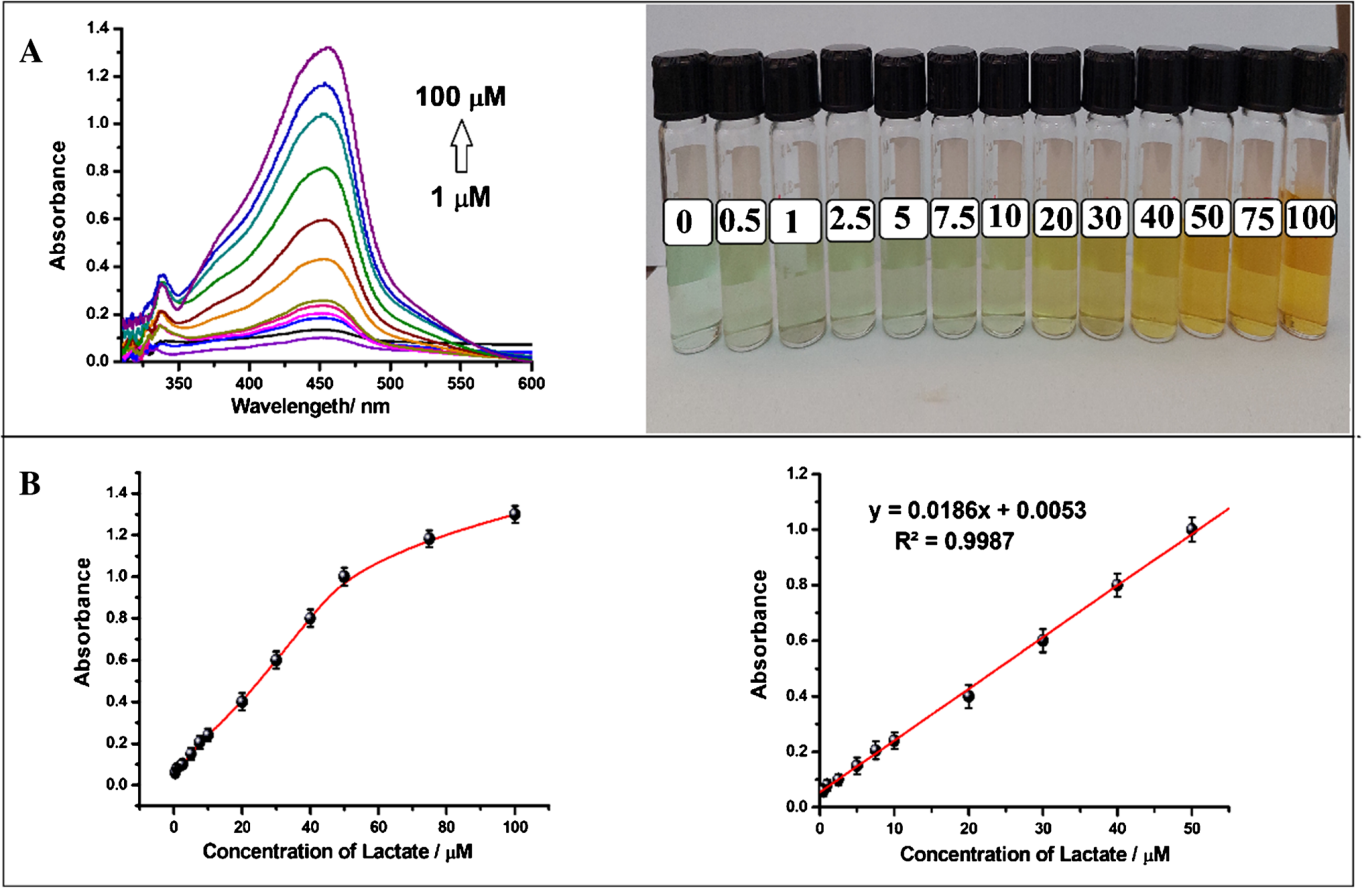
**A** Spectra and photographs recorded for lactate biosensor with increasing lactate concentration using bienzyme-immobilized nanoparticles (LOx-SiO_2_@Fe_3_O_4_ + POx-SiO_2_@Fe_3_O_4_). **B** The curve of absorbance recorded at 450 nm versus lactate concentration and linear calibration plot

Optimal conditions were used to carry out enzymatic reactions with various lactate concentrations ranging from 1 to 150 µM for the monoenzyme (LOx-SiO_2_@Fe_3_O_4_) and from 0.5 to 100 µM for the bienzyme (LOx-SiO_2_@Fe_3_O_4_ + POx-SiO_2_@Fe_3_O_4_)-based biosensor. The absorbance of the [Cu(Nc)_2_]^+^ complex, created by adding the CUPRAC reagent after the enzyme reaction, was measured at 450 nm. The recorded photographic images and spectra together with the calibration curves are given in Figs. 2 and 3, respectively. The calibration plot obtained from the monoenzyme had linearity between 1 and 100 µM (Fig. 2), while that obtained from the bienzyme was linear between 0.5 and 50 µM (Fig. 3). To evaluate the precision of the designed bienzymatic lactate biosensor, three different calibration studies were carried out. Using the slope obtained from the calibration curves, the RSD was found to be 3.8%, reflecting the good repeatability of the proposed biosensor. In addition, the LOD value (0.17 µM) obtained with the bienzyme was found to be approximately two times lower than that obtained with the monoenzyme (0.37 µM). Furthermore, when the slopes obtained from the linear calibration curve are compared, it is seen that the analytical sensitivity of the bienzymatic biosensor (slope is 0.0186 L/µmol.cm) has increased about 1.6-fold compared to that of monoenzymatic biosensor (0.0118 L/µmol.cm). Moreover, the slope of the monoenzymatic lactate biosensor was found to be very close to that obtained for the lactate biosensor using the enzyme in solution media (0.0107) and also to that obtained for pure H_2_O_2_ (0.0126). This suggests that the designed lactate biosensor performs comparably to other existing biosensors and demonstrates its potential for sensitive lactate detection. The close similarity in slopes further validates the reliability and effectiveness of the designed biosensor in measuring lactate levels.

A comparison of the analytical performance of the proposed sensing method with some previously reported colorimetric, fluorimetric, and even electrochemical lactate biosensors is given in Table 1. It can be seen that different colorimetric procedures, such as (i) the formation of TMB_ox_ or ABTS with enzymatically produced H_2_O_2_ in the presence of LOx and HRP, (ii) using 4-aminoantipyrine and *N*-ethyl-*N*-(2-hydroxy-3-sulfopropyl)-m-toluidine as a chromogenic dye with LOx, (iii) etching of bimetallic nanoparticles based on the LOx enzyme, and (iv) the formation of formazan dye from WST-8 with enzymatically produced NADH based on LDH, have been exploited in the design of lactate biosensors. The LOD value of the proposed CUPRAC method is lower than those of most similar colorimetric methods, which reflects the higher sensitivity of the proposed method than those of analogous methods. The LOD value of this work is only competitive with that of etching of plasmonic bimetallic nanoparticles. In addition, the LOD value of the proposed CUPRAC method was also found to be lower or more competitive than that of the fluorimetric and electrochemical methods. Moreover, when compared to all these methods, it was observed that the linearity range of the new CUPRAC-LOx- and POx-based biosensors was wider than those of most studies, even though some studies came closer. The wideness of the linear range, which normally covers one order of magnitude, can be explained by the high molar extinction coefficient and, in particular, the high equilibrium constant of the reaction between enzymatically produced H_2_O_2_ and the CUPRAC reagent to give rise to a single light-absorbing product, i.e., cuprous-neocuproine: Cu(Nc)_2_^+^. Hence, the magnitude of the equilibrium constant indicates the stability of the product formed and leads to the minimization of deviations from Beer’s Law and the widening of the linear range due to single-product chemistry denoted by the reaction equation:

$$2\;{\mathrm{Cu}(\mathrm{Nc})}_2^{2+}+{\mathrm H}_2{\mathrm O}_2\rightarrow2\;{\mathrm{Cu}(\mathrm{Nc})}_2^++{\mathrm O}_2+{2\;\mathrm H}^+$$

where the liberated protons are neutralized by the ammonium acetate buffer component of the CUPRAC reagent. As we mentioned in our previous glucose biosensor study [35], while the proposed colorimetric reaction between [Cu(Nc)_2_]^2+^ and NADH has a definite stoichiometry, the color reaction due to the oxidation of TMB in a H_2_O_2_ environment does not have a clear stoichiometry. The reason is that H_2_O_2_ degrades into a number of reactive oxygen species (ROS) in the presence of peroxidases or their mimics, and even molecular oxygen behaves as an oxidant toward TMB, producing an indefinite stoichiometry for oxidized TMB formation and a relatively poor linearity of analyte concentration dependence [55]. Either HRP enzyme or peroxidase mimic nanoparticles are needed for the TMB-based colorimetric reaction to occur. Thus, it may be concluded that this newly designed bienzymatic lactate biosensor has advantages over other analogous biosensors in the literature such as ease of use, simplicity, definite reaction stoichiometry, higher sensitivity, and ability to remove important interferences with pre-oxidants and that this sensor is predicted to find use in diverse areas.

**Table 1:** Comparison of the analytical performance of the constructed biosensor with those of related optical biosensors

Method	Procedure	Linear range (mM)	LOD (mM)	Sample	Ref
Colorimetric	LOx- and POx-immobilized SiO_2_@Fe_3_O_4_ + CUPRAC reagent as chromogenic oxidant. Absorbance measured at 450 nm	5 × 10^−4^–5 × 10^−2^	1.7 × 10^−4^	Real and artificial sweat, artificial serum, milk	*This work*
	Au–Ag/C NC + LOx based on the etching of bimetallic Nps. Absorbance measured at 420 nm	1 × 10^−4^–2.2 × 10^−2^, 2.2 × 10^−2^–0.22	3.3 × 10^−5^	Serum	[52]
Smartphone colorimetric	LOx/HRP with TMB immobilized on a TNT/alginate scaffold integrated on paper-based sensing platform	0.1–1.0	0.069	Real sweat	[56]
	LOx/HRP/TMB using paper-based wearable platform	0–1.0	0.06	Real sweat	[45]
	LOx-immobilized polystyrene microwells and paper-based biosensors using 4-aminoantipyrine, and *N*-ethyl-*N*-(2-hydroxy-3-sulfopropyl)-m-toluidine as the color reagent	0.5–3.0	–	Artificial blood	[57]
	LDH-CBD paper + NAD^+^ + WST-8 based on formation of formazan	0.5–8.0	^–^	–	[27]
	LOx + HRP and microfluidic textile-based biosensor using ABTS	0–11	^–^	Real sweat	[26]
Fluorimetric	LOx/Cat/PdTCpp-based composite hydrogels	~ 4.5 × 10^−3^–0.83	–	–	[58]
	LOx + TA + CuO NPs based on florescence TA which oxidized OH. Formed after reaction between enzymatically produced H_2_O_2_ and CuO NPs	5 × 10^−3^–0.2	3.4 × 10^−4^	Human serum	[53]
	AgNPs coated carbon quantum dots + LOx based on florescence of CDs after etching of AgNPs with enzymatically produced H_2_O_2_	0.02–18	0.01	4T1 cells	[59]
Electrochemical	Au/rGO/PtNps/LOx/Nf based on chronoamperometry recorded at 0.5 V in pH 7.4 PBS	0–10	2.04 × 10^−3^	Artificial interstitial fluid and human serum	[60]
	LOx/GO-ZnO/SPCE based on chronoamperometry recorded at 0.4 V in pH 7.4 PBS	0.015–1.25	9.0 × 10^−3^	Saliva	[61]
	PU/LOx/PANI/m-PD/SPAuE based on chronoamperometry recorded at 0.7 V in pH 7.4 PBS	0.2–5	7.9 × 10^−3^	Human blood plasma	[62]

*LOx* lactate oxidase, *POx* pyruvate oxidase, *SiO*_*2*_*@Fe*_*3*_*O*_*4*_ silanized magnetite nanoparticles, *CUPRAC* cupric reducing antioxidant capacity, *HRP* horseradish peroxidase, *TMB* 3,3′,5,5′-tetramethylbenzidine, *TNT* trinitrotoluene, *LDH* lactate dehydrogenase, *CBD* cellulose binding domain, *NAD*^+^ β-nicotinamide adenine dinucleotide, *WST-8* tetrazolium salt, *Au–Ag/C NC* carbon-based gold core silver shell Au–Ag bimetallic nanocomposite, *Cat* Catalase, *PdTCPP* Pd-meso-tetra (4-carboxyphenyl) porphyrin, *TA* terephthalic acid, *CuO NPs* cupric oxide nanoparticles, *AgNPs* silver nanoparticles, *CD* carbon dots, *rGO* reduced graphene oxide, *PtNPs* platinum nanoparticles, *Nf* Nafion, *ZnO* zinc oxide, *GO* graphene oxide, *SPCE* screen-printed carbon electrode, *PU* polyurethane, *PANI* polyaniline, *m-PD* m-phenylenediamine, *SPAuE* screen-printed gold electrode

### Mechanism of the CUPRAC reagent-based colorimetric lactate biosensor

**Fig. 4:**
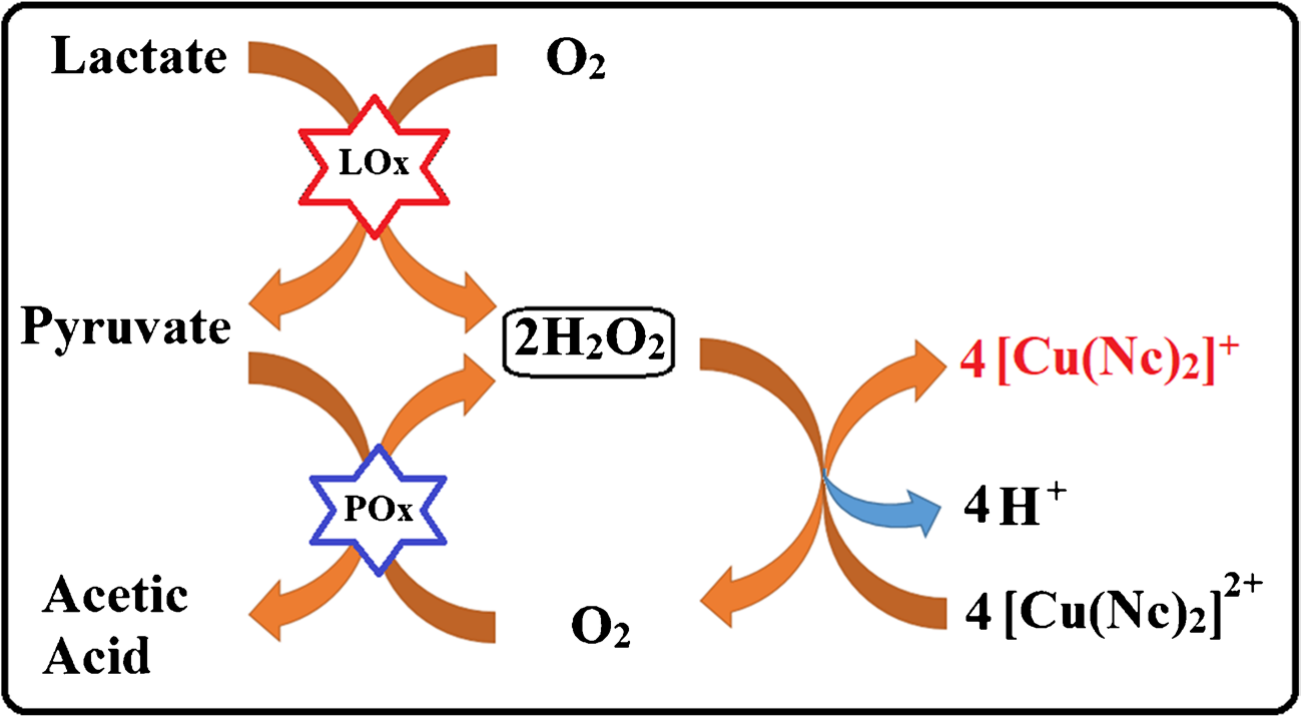
Schematic representation of lactate biosensor based on using bienzyme-immobilized magnetite nanoparticles (LOx-SiO_2_@Fe_3_O_4_ + POx-SiO_2_@Fe_3_O_4_)

The mechanism of lactate biosensors can be explained with the aid of two simultaneous enzymatic and one sequential colorimetric reaction. The first enzymatic reaction takes place between SiO_2_@Fe_3_O_4_-bound LOx and lactate in solution in the presence of molecular O_2_. At the end of this reaction, lactate is oxidized to pyruvate, while O_2_ is reduced to H_2_O_2_. Subsequently, a second enzymatic reaction immediately occurs between pyruvate and SiO_2_@Fe_3_O_4_-bound POx, in which pyruvate is oxidized to acetic acid and molecular O_2_ is reduced to H_2_O_2_. After separating enzyme-immobilized SiO_2_@Fe_3_O_4_ nanoparticles using a magnet, a colorimetric reaction takes place between 4 mol of the CUPRAC reagent (i.e., cupric-neocuproine) and a total of 2 mol H_2_O_2_ produced enzymatically per 1 mol of the substrate. Thus, the lactate biosensor is established based on measuring the absorbance at 450 nm of the yellow-orange [Cu(Nc)_2_]^+^ complex formed proportionally with lactate concentration, and the sensitivity of the bienzyme-immobilized sensor is double-fold increased with respect to that of the single enzyme sensor. The operational mechanism of the bienzymatic biosensor is represented in Fig. 4.

### Interference studies

During the interference studies with the designed biosensor, possible interfering molecules such as ascorbic acid (AA), uric acid (UA), dopamine (DA), and some mono- and disaccharides (sucrose, galactose, fructose, lactose, maltose) were tested. Firstly, interferents were prepared at 50 µM, and their interactions with CUPRAC reagent (750 µL 1.0 M NH_4_CH_3_COO + 500 µL Cu(II) + 750 µL Neocuproine + (500 − *x*) µL purified water) were investigated. The results showed that glucose, fructose, maltose, galactose, sucrose, and lactose did not give any yellow color with the CUPRAC reagent. In contrast, the interfering substances DA, UA, and AA reacted with the CUPRAC reagent to form a yellow-orange [Cu(Nc)_2_]^+^ complex. The reducing agents, DA, UA, and AA (especially DA being a stronger interferent than others), positively interfere with enzymatic biosensors. In our previously constructed glucose biosensor based on dehydrogenase enzyme and NAD^+^, it was reported that the use of a pre-oxidant (NaBiO_3_, a strong oxidant) significantly attenuated the interference of these substances before the enzymatic and colorimetric reactions [35, 63]. Thus, before the enzymatic reaction followed by interaction with the CUPRAC reagent, the solutions were passed through a syringe containing 0.5 g NaBiO_3_ to eliminate these interferences. Enzymatic reactions were then carried out using LOx- and POx-immobilized magnetic nanoparticles, followed by recording colorimetric responses with the addition of CUPRAC. It was determined from the absorbance values obtained that the interfering compounds were reduced and pre-eliminated to yield the results in Table 2.

**Table 2:** Results obtained from interference study for lactate biosensor using bienzyme immobilized magnetite nanoparticles (LOx-SiO_2_@Fe_3_O_4_ + POx-SiO_2_@Fe_3_O_4_)

Molecules	A_450_ after only CR of 50 µM of species		Analyte-to-interfent ratio	ER (30 min) + CR (5 min)		Interference %	
	Without NaBiO_3_	With NaBiO_3_	L = 25 µM Interferences: 50 (for 1:2) or 5 µM (for 5:1)	Without NaBiO_3_	With NaBiO_3_	Without NaBiO_3_	With NaBiO_3_
Lactate (L) (analyte)	0.025 ± 0.007	–	L	0.612 ± 0.015	0.602 ± 0.017	–	–
Fructose (F)	0.004 ± 0.002	–	1:2 L:F	0.635 ± 0.012	–	–	–
Maltose (M)	0.019 ± 0.004	–	1:2 L:M	0.622 ± 0.015	–	–	–
Galactose (G)	0.007 ± 0.003	–	1:2 L:G	0.614 ± 0.017	–	–	–
Lactose (LA)	0.011 ± 0.001	–	1:2 L:LA	0.605 ± 0.025	–	–	–
Sucrose (S)	0.009 ± 0.006	–	1:2 L:S	0.627 ± 0.026	–	–	–
DA	2.105 ± 0.042	0.017 ± 0.003	1:2 L:DA	2.137 ± 0.023	0.636 ± 0.013	+ 249.2	+ 5.6
			5:1 L:DA	0.972 ± 0.022	0.632 ± 0.019	+ 58.8	+ 5.0
AA	0.813 ± 0.008	0.027 ± 0.009	1:2 L:AA	1.121 ± 0.016	0.605 ± 0.023	+ 83.2	+ 5.0
			5:1 L:AA	0.884 ± 0.031	0.591 ± 0.021	+ 44.4	− 1.8
UA	1.129 ± 0.032	0.031 ± 0.011	1:2 L:UA	1.158 ± 0.024	0.651 ± 0.018	+ 89.2	+ 8.1
			5:1 L:UA	0.816 ± 0.010	0.660 ± 0,024	+ 33.3	+ 9.6
DA + AA + UA	2.157 ± 0.072	0.151 ± 0.008	5:1:1:1 L:DA:AA:UA	1.126 ± 0.011	0.600 ± 0.015	+ 84.0	− 0.3

### Stability studies

**Fig. 5:**
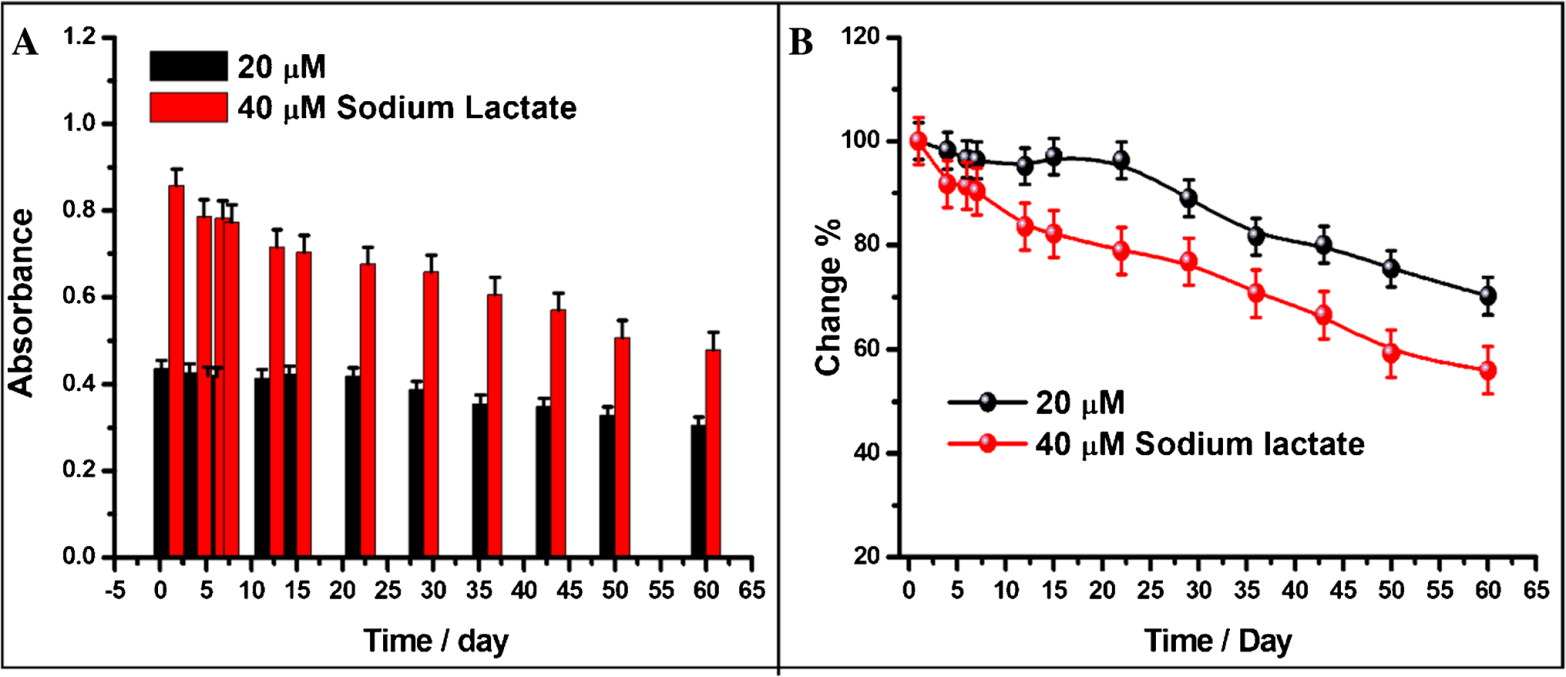
Absorbance-time bar diagram (**A**) and percentage change-time graph (**B**) for two distinct concentrations of lactate with the use of lactate biosensor using LOx-SiO_2_@Fe_3_O_4_ + POx-SiO_2_@Fe_3_O_4_ nanoparticles

Storage stability studies of the fabricated lactate biosensor were carried out by keeping the mixture of LOx-SiO_2_@Fe_3_O_4_ + POx-SiO_2_@Fe_3_O_4_ nanoparticles slightly moist in pH 5.0 PBS buffer (0.10 M) at + 4 °C in a closed bottle and recording absorbance values for lactate at two different concentrations once a week. The lactate biosensors based on LOx and POx enzymes yielded consistent results for approximately 1 month. This might arise from the fact that the chitosan film is very stable in silica-coated Fe_3_O_4_, the environment is biocompatible, and the enzyme is successfully immobilized, which prevents any enzyme leakage. Based on the findings from the 2-month study, it is evident that the developed biosensor demonstrates higher stability in detecting low concentrations. Figure 5 displays the absorbance-time bar diagram and the percentage change in absorbance (relative to the absorbance value on the first day) versus the time graph.

### Real sample analysis

In accordance with spiking protocols in real samples, all samples were selected so as to contain an initial amount of analyte (lactate). In real sample studies, 40 µL of artificial blood, 10 µL of artificial sweat, and 50 µL of real sweat sample diluted 10 times with 1.0 M NH_4_CH_3_COO samples were added to 750 µL pH 7.0 buffer solution, and enzymatic reactions were carried out using the mixture of LOx-SiO_2_@Fe_3_O_4_ and POx-SiO_2_@Fe_3_O_4_ nanoparticles. Then CUPRAC reagent was added to the magnetically separated solutions to obtain a 2.5 mL final volume, and the amount of lactate was determined by measuring the absorbance values at 450 nm after the colorimetric reaction. Then, a real sweat sample was spiked with 5 mM and 10 mM sodium lactate. The spiked samples of 35 µL (for 5 mM) and 25 µL (10 mM) of the 10 times diluted with 1.0 M NH_4_CH_3_COO were used for the enzymatic reaction, and the final volume was fixed at 2.5 mL by the colorimetric reaction. Following enzymatic and colorimetric processes, absorbance values were recorded, and recovery values were calculated. To determine the lactate content of cow milk, three drops of concentrated HCl were added to 5.0 mL of cow milk, and the milk proteins precipitated at 60 °C. The solution was then centrifuged at 4000 rpm for 15 min. The supernatant phase was used before the enzymatic reaction by passing it through a syringe filled with 0.5 g of NaBiO_3_. After adding 750 µL of 1 M NH_4_CH_3_COO to 65 µL of unspiked milk, the enzymatic reaction was carried out with LOx-SiO_2_@Fe_3_O_4_ + POx-SiO_2_@Fe_3_O_4_ nanoparticles under optimized conditions. After the separation of the magnetic nanoparticles with a magnet, CUPRAC reagent was added to the samples until a 2.5 mL final volume was obtained, the colorimetric reaction was performed, and absorbance values at 450 nm were measured. Subsequently, 1.0 mM and 1.5 mM sodium lactate were spiked into 5.0 mL of milk sample, and the above precipitation procedures were repeated. In this case, 35 µL and 25 µL milk samples spiked with 1.0- and 1.5-mM lactate, respectively, were used for the enzymatic reaction, and the final volume was fixed at 2.5 mL by the colorimetric reaction. Absorbance values after the enzymatic and colorimetric reactions were recorded, and recovery values were subsequently calculated. All results are given in Table 3.

**Table 3:** Analysis of real samples using the proposed optical biosensor

Sample	Added lactate (mM)	Found lactate concentration (mM)	%Recovery
Milk	0	0.95 ± 0.02	–
	1.00	1.96 ± 0.05	100.5
	1.50	2.46 ± 0.07	100.4
Artificial blood serum (containing 1.5 mM*)	0	1.53 ± 0.04	102.0
Artificial sweat serum (5.5 mM*)	0	5.64 ± 0.05	102.5
Real sweat	0	10.73 ± 0.22	–
	5.00	15.36 ± 0.35	97.6
	10.00	21.31 ± 0.41	102.8

*Lactate concentration added to artificial blood and sweat samples

## Conclusions

In this study, a successful and novel colorimetric lactate sensor was developed using the CUPRAC reagent as a chromogenic oxidant and a mixture of LOx- and POx-immobilized magnetite nanoparticles (LOx-SiO_2_@Fe_3_O_4_ and POx-SiO_2_@Fe_3_O_4_) for the first time. The enzyme–substrate reaction liberated H_2_O_2_, capable of reducing Cu(II)-neocuproine to the highly colored Cu(I)-neocuproine chelate. The absorbance value recorded at 450 nm based on the formation of the [Cu(Nc)_2_]^+^ complex in the developed biosensor increased proportionally with the lactate concentration in the range of 0.5 to 50 μM with the LOD of 0.17 µM. Aside from the enhanced substrate selectivity of the binary enzyme–immobilized sensor, this bienzyme biosensor gave sensitive responses that were twice those of the monoenzyme. The fact that 2 mol of H_2_O_2_ are liberated at bienzymatic reactions per 1 mol of the substrate means that more H_2_O_2_ reacts with the CUPRAC reagent, resulting in higher color intensity and higher sensitivity than that produced by monoenzyme for the same concentration of lactate. The designed sensor has a clear stoichiometry for the oxidation of H_2_O_2_ to molecular oxygen with cupric-neocuproine, as opposed to the indefinite stoichiometries for the oxidation of redox dyes like TMB with H_2_O_2_ and its degradation products (ROS) in the presence of peroxidases or their mimics. Interference studies show that DA, AA, and UA give significant positive interference on the manufactured biosensor due to their high tendency to chemically reduce the CUPRAC reagent. However, these interferences were significantly eliminated by using NaBiO_3_ as a pre-oxidant before the substrate-specific colorimetric reaction. The manufactured biosensor demonstrated acceptable stability and remained at satisfactory performance for approximately 1 month. The chitosan film in silica-coated Fe_3_O_4_ exhibits exceptional stability, which contributes to the overall long-term stability for at least 1 month. The biocompatible environment and effective immobilization of the enzyme further increase this stability by preventing any enzyme leakage. The designed bienzymatic colorimetric biosensor was successfully applied to both simulated and real samples originally containing lactate. Recoveries close to 100% were obtained in the lactate-spiked real sweat and milk samples, which reflects the high accuracy of the designed biosensor.

### Supplementary Information

Below is the link to the electronic supplementary material.

Supplementary file1 (DOCX 2967 KB)

## Data Availability

The data generated and analyzed during this study are included in the article and in the supplementary information file. In addition, any data requested from the corresponding author can be provided upon reasonable request.
