# Supplementary material for: A new colorimetric lactate biosensor based on CUPRAC reagent using binary enzyme (lactate-pyruvate oxidases)-immobilized silanized magnetite nanoparticles
Source: Mikrochim Acta. 2024 Jul 9;191(8):455. doi: 10.1007/s00604-024-06531-w (PMC11233342; doi:10.1007/s00604-024-06531-w)
Supplement: Supplementary file 1 — Supplementary file1 (DOCX 2967 KB) [file 604_2024_6531_MOESM1_ESM.docx]

**Supplementary Information**

**A New Colorimetric Lactate Biosensor Based on CUPRAC Reagent Using Binary Enzyme (Lactate-Pyruvate Oxidases)−Immobilized Silanized Magnetite Nanoparticles**

Selen Ayaz^1^, Teslime Erşan^1^, Yusuf Dilgin^1^*, Reşat Apak^2^*

^1^Çanakkale Onsekiz Mart University, Faculty of Science, Department of Chemistry, Canakkale, Türkiye

^2^İstanbul University-Cerrahpaşa, Faculty of Engineering, Department of Chemistry, İstanbul-Avcılar, Türkiye

Note for using the CUPRAC Reagent: Colorimetric reagents were prepared by mixing solutions prepared from both Cu (II) salt (CuCl_2_) and pure neocuproine (Nc). The thermodynamic stability constants of the 1:2 complexes of Cu(II)-Nc and Cu(I)-Nc are 10^12^ and 10^19^, respectively, which means that once the neocuproine ligand enters the coordination sphere of Cu(II), the existing chloride ions and weakly basic water molecules will be easily displaced by neocuproine molecules due to the large equilibrium constant of cupric-neocuproine, giving rise to the distorted tetrahedral complex of [Cu(Nc)_2_]^2+^. Therefore, since the complex is formed with ML_2_ (metal:M, ligand:L) stoichiometry, excess Cu(II) or anionic species in the environment have no effect on the colorimetric reaction. As a result, the complex was prepared in pure form by mixing pure metal ion and ligand. Therefore, it is not practical to first synthesize the CUPRAC reagent and purify it before use, which may unnecessarily involve long and tedious synthesis and characterization steps. Moreover, such a procedure will not affect the sensitivity or selectivity of the colorimetric reaction.


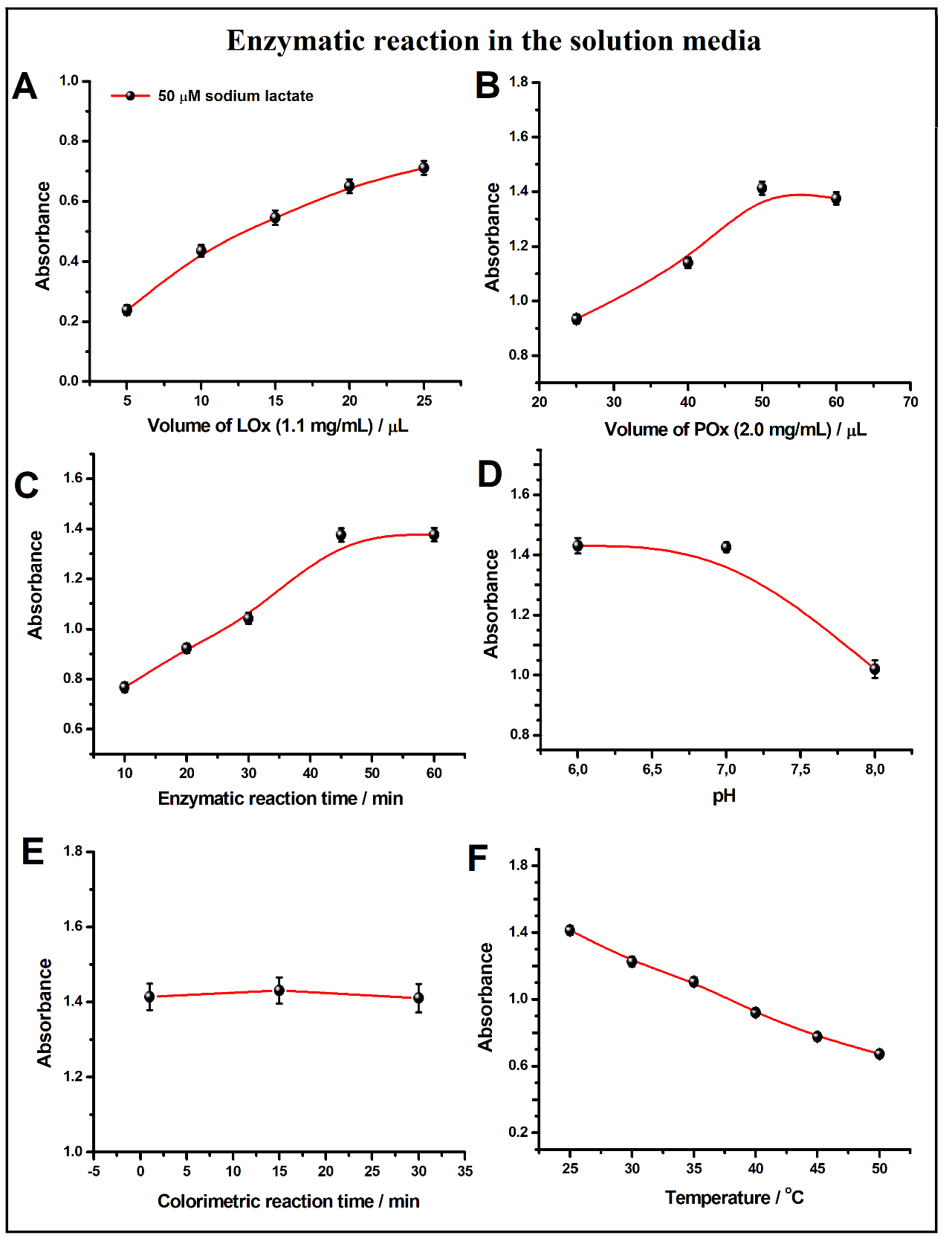


**Fig. S1.** Optimization curves of **A)** enzyme amount of LOx in the absence of POx, **B)** enzyme amount of POx in the presence of 25 µL of 1.1 mg/mL LOx and following optimization curves of **C)** enzymatic reaction time, **D)** pH, **E)** colorimetric reaction time and **F)** temperature using bienzyme (25 µL of 1.1 mg/mL LOx+ 50 µL of 2.0 mg/mL POx used in the presence of solution media)


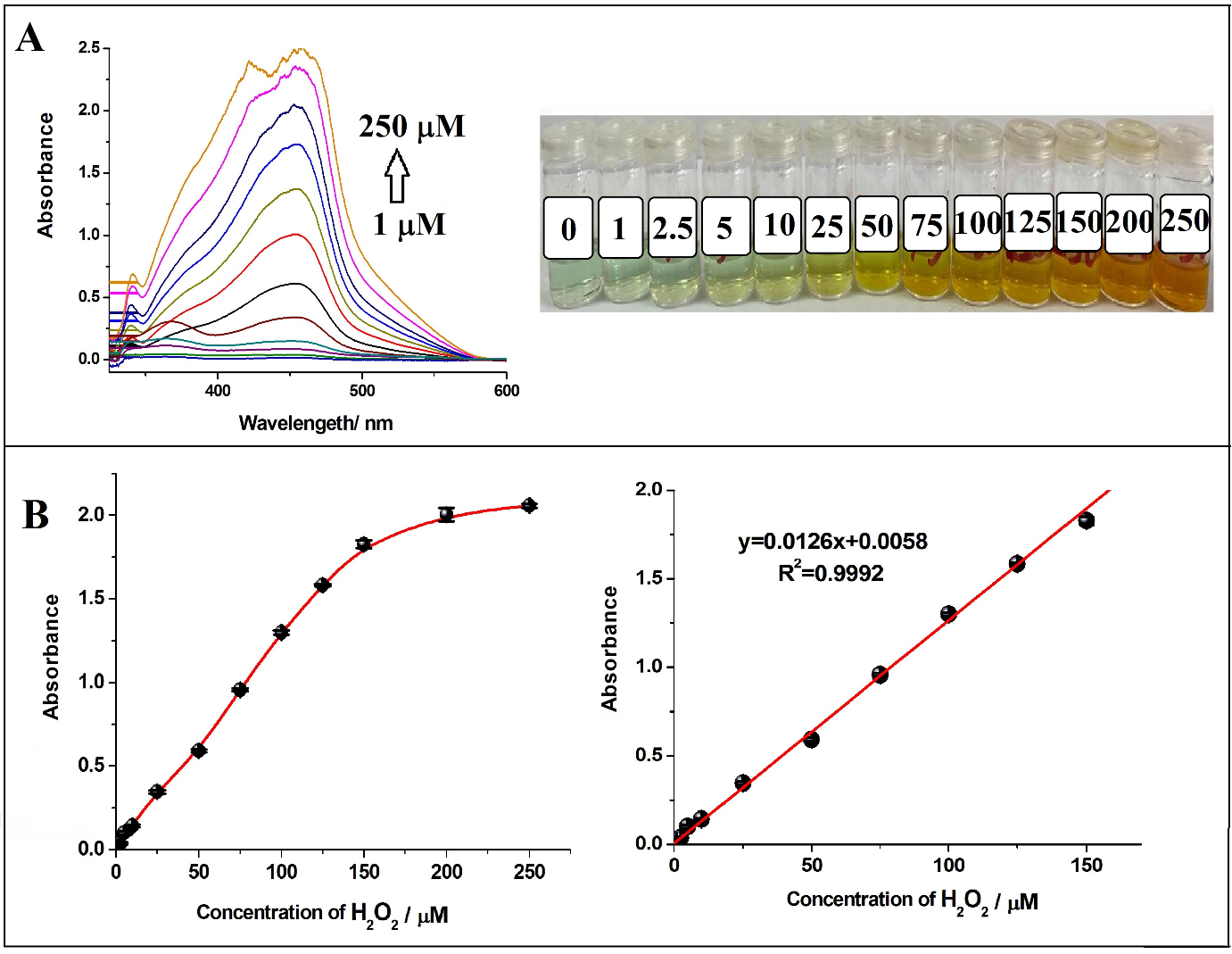


**Fig S2. A)** Spectra and photographs of of [Cu(Nc)_2_]^+^ formed as a result of the reaction between [Cu(Nc)_2_]^2+^ and pure H_2_O_2_ (production from enzymatic reaction **B)** The curve of absorbance recorded at 450 nm *versus* H_2_O_2_ concentration, and linear calibration plot.


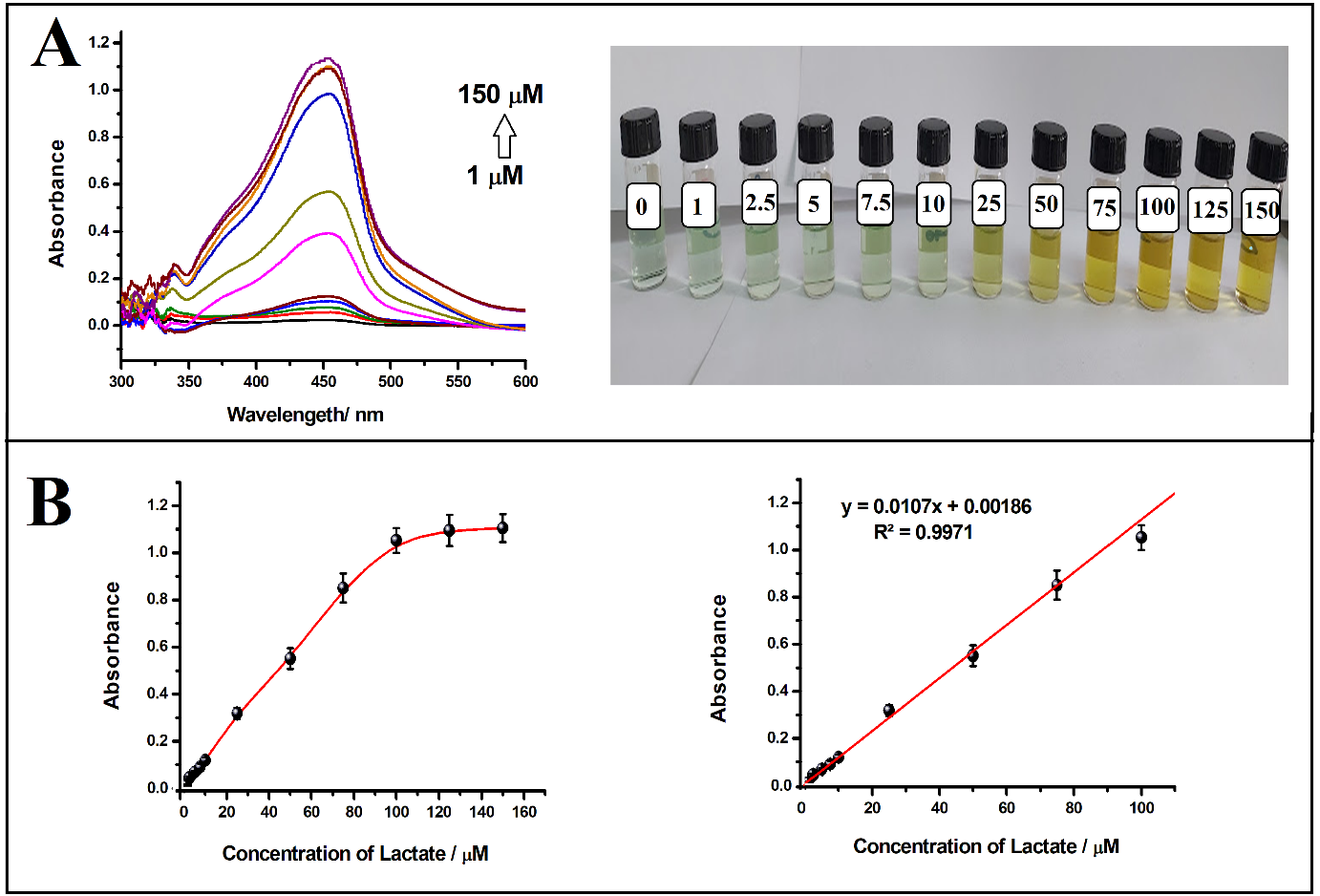


**Fig. S3.** **A)** Spectra and photographs of [Cu(Nc)_2_]^+^ formed as a result of the reaction between [Cu(Nc)_2_]^2+^ and H_2_O_2_, which was formed by the enzymatic reaction of LOx (25 µL of 1.1 mg/mL enzyme) used in the solution media (without immobilization of enzyme) with different concentrations of lactate **B)** The curve of absorbance recorded at 450 nm versus H_2_O_2_ concentration, and linear calibration plot.

FTIR spectra of bare, silanizated and mono- and bi-enzyme immobilized magnetite nanoparticles are shown in **Fig. S4.** The O-H stretching and bending vibrations due to adsorbed water on bare Fe_3_O_4_ NPs were observed at 1634 cm^-1^ and 3400 cm^-1^ respectively **(Fig. S4/a).** Moreover, peaks at 1040 cm^-1^ and 800 cm^-1^ attributed to characteristic symmetric and asymmetric Si-O-Si stretching vibrations and a sharp peak at 955 cm^-1^ attributed to Si-OH stretching vibration proves the silanization of magnetite nanoparticles with reagents of APTES- and TEOS **(Fig. S4/b).** When monoenzyme (LOx) **(Fig. S4/c)** and bienzyme (LOx+POx) **(Fig. S4/d)** were immobilized onto chitosan (CS) coated SiO_2_@Fe_3_O_4_ via crosslinking with glutaraldehyde (GA), the characteristic carbonyl stretching at 1600 cm^-1^ became more pronounced; the peak at 1410 cm^-1^ seen in unimmoblized nanoaprticles (b) shifted to a more positive region by approximately 50 cm^-1^(c and d), and two small new peaks were observed at 1390 and 1300 cm^-1^, respectively. Similar results were also obtained in our previous study in which GDH was immobilized on SiO_2_@Fe_3_O_4_ NPs [1] and in the literature for the immobilization of LOx and POx [2-3]. All these results prove that monoenzyme (LOx) and bienzyme (LOx + POx) are effectively immobilized on magnetite nanoparticle surfaces.


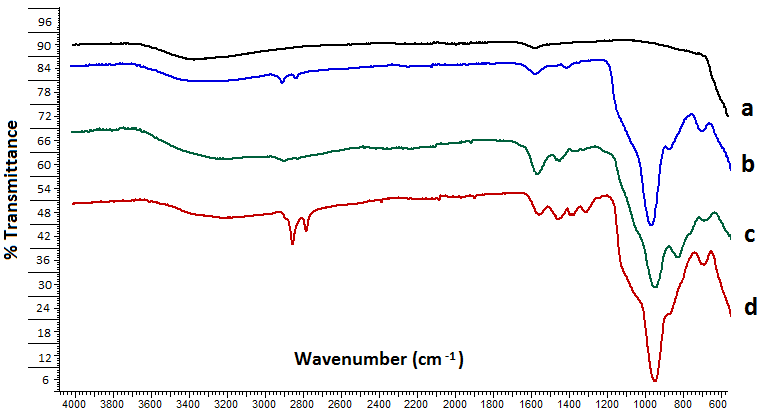


**Fig. S4.** FTIR spectra of **a)** bare Fe_3_O_4_, **b)** SiO_2_@Fe_3_O_4_, **c)** LOx-CS@SiO_2_@Fe_3_O_4_ NPs and **d)** (LOx+POx)-CS@SiO_2_@Fe_3_O_4_ NPs

Fig. S5 shows SEM images of bare Fe_3_O_4_, SiO_2_@Fe_3_O_4_, and LOx+POx-CS@SiO_2_@Fe_3_O_4_. It can be seen that uncoated Fe_3_O_4_ NPs are in the form of rough sand particles(Fig. S5A). However, silanization with TEOS and APTES resulted in a more homogeneous and rough spherical structure (Fig. S5B). Then, the morphology of the surface was further changed by the immobilization of the bienzyme (LOx+POx). Moreover, changes in Si atoms’ atomic percent in the EDX spectrum (Fig. S6) and color distribution in elemental mapping (Fig. S7) indicated that Fe_3_O_4_ NPs were coated with the silanization reagents of TEOS and APTES. Following this, the increasing C% and N% support the immobilization of enzymes due to the amino acids in the enzyme structure. The elemental mapping dependent on considering only Si and N atoms (Fig. S8) shows that 9% of N atoms in SiO_2_@Fe_3_O_4_ increased up to 12% with immobilization of bienzymes.


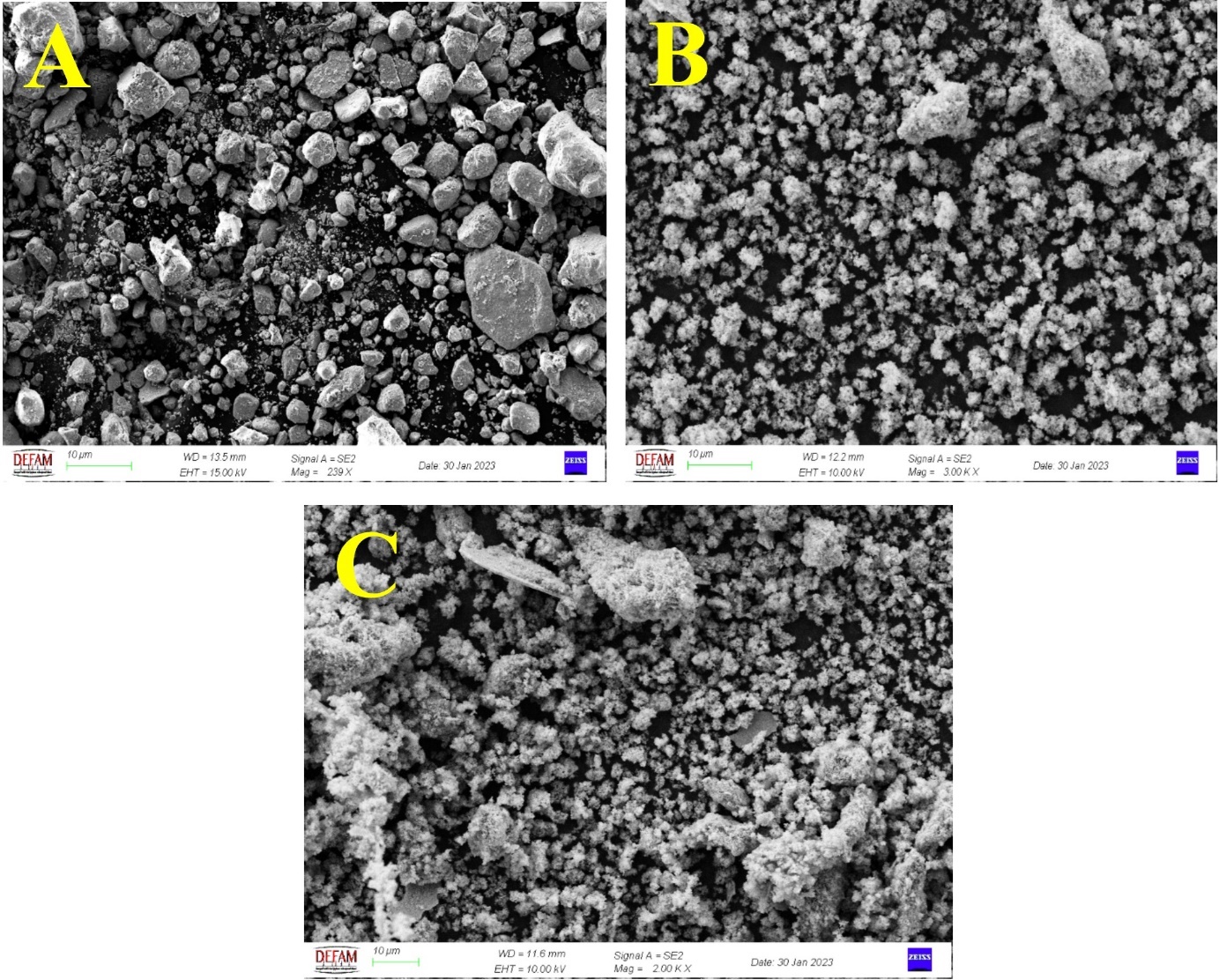


**Fig. S5.** SEM images of **A)** bare Fe_3_O_4_, **B)** SiO_2_@Fe_3_O_4_, and **C** (LOx+POx)-CS@SiO_2_@Fe_3_O_4_ NPs


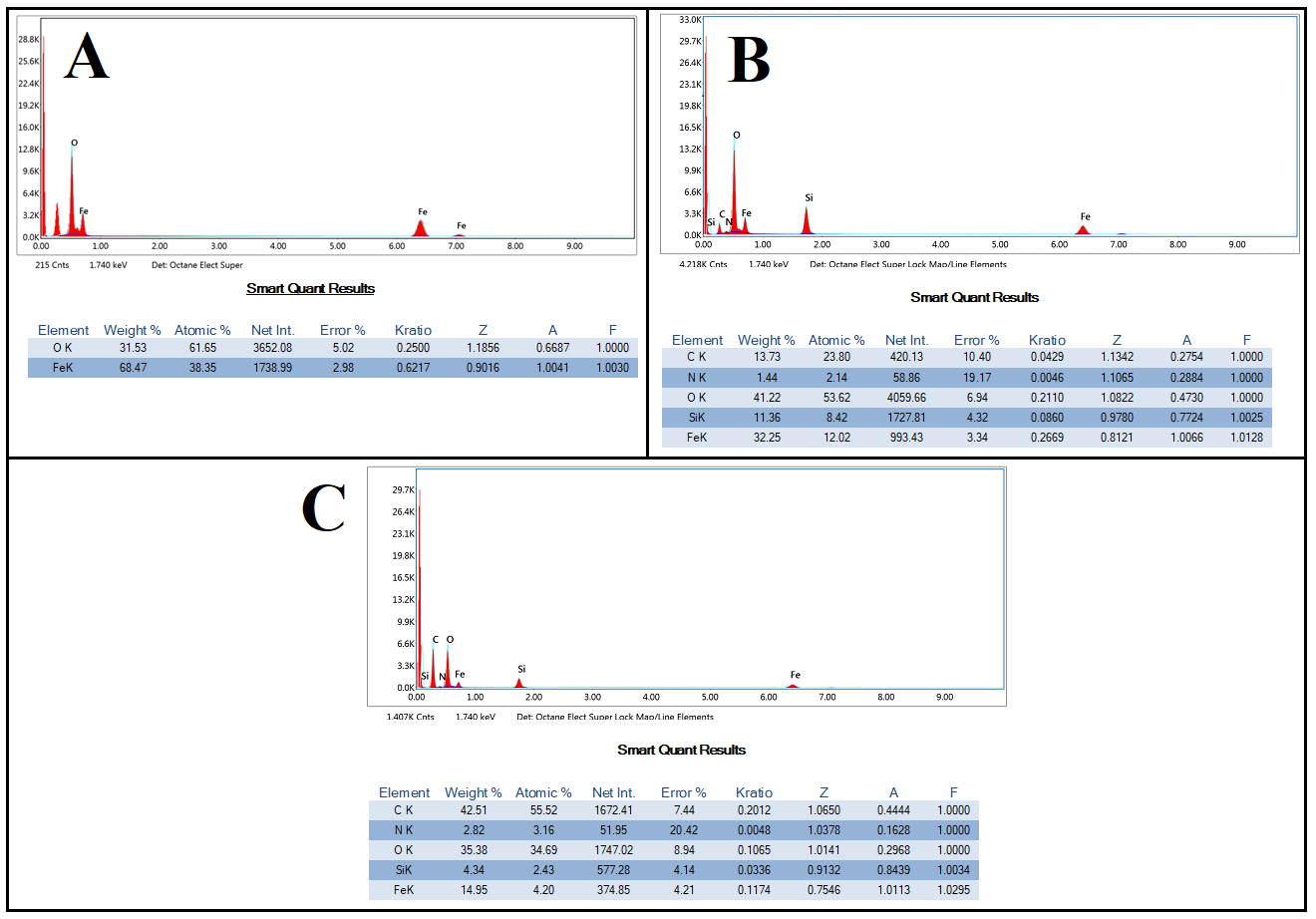


**Fig. S6.** EDX spectra of **A)** bare Fe_3_O_4_, **B)** SiO_2_@Fe_3_O_4_, and **C** (LOx+POx)-CS@SiO_2_@Fe_3_O_4_ NPs


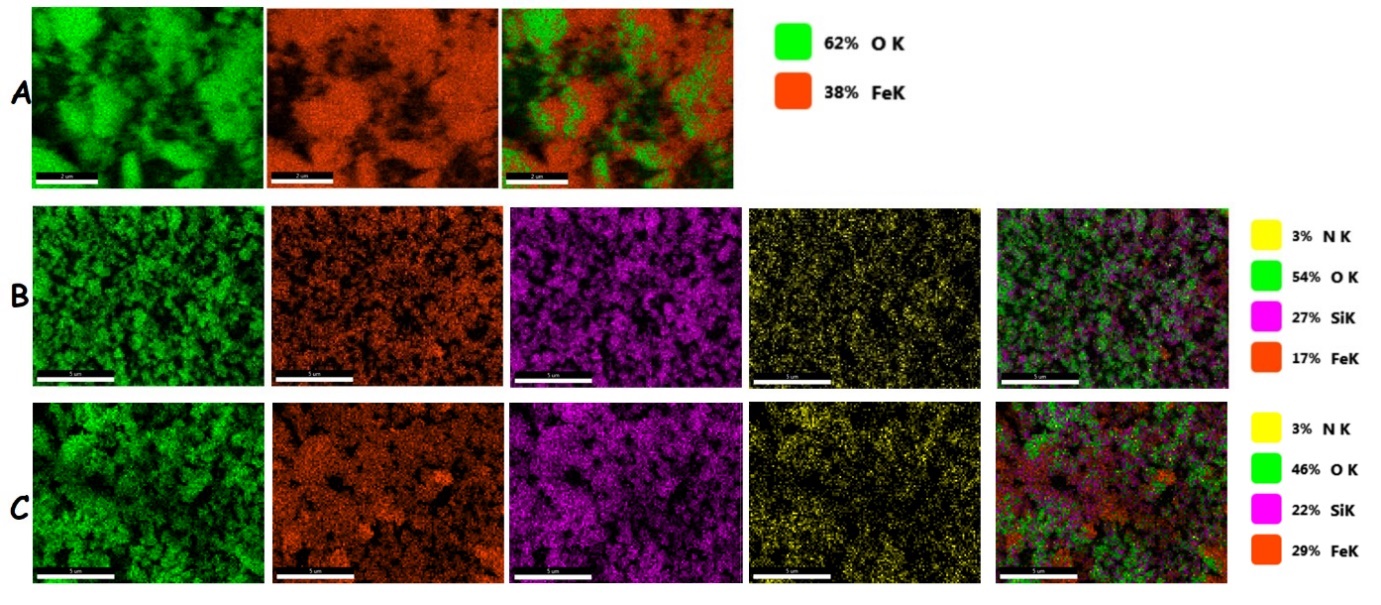


**Fig. S7.** Elemental mapping of **A)** bare Fe_3_O_4_, **B)** SiO_2_@Fe_3_O_4_, and **C** (LOx+POx)-CS@SiO_2_@Fe_3_O_4_ NPs


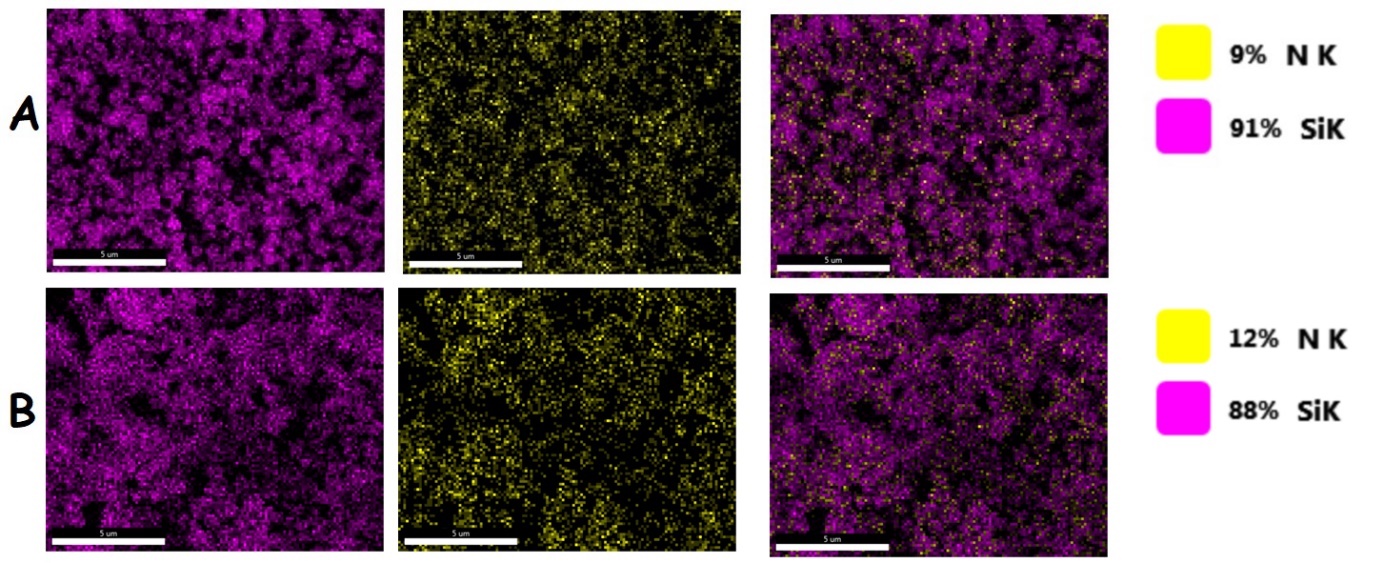


**Fig. S8.** Elemental mapping of **A)** SiO_2_@Fe_3_O_4_, and **B** (LOx+POx)-CS@SiO_2_@Fe_3_O_4_ NPs recorded considering Si and N atoms

**
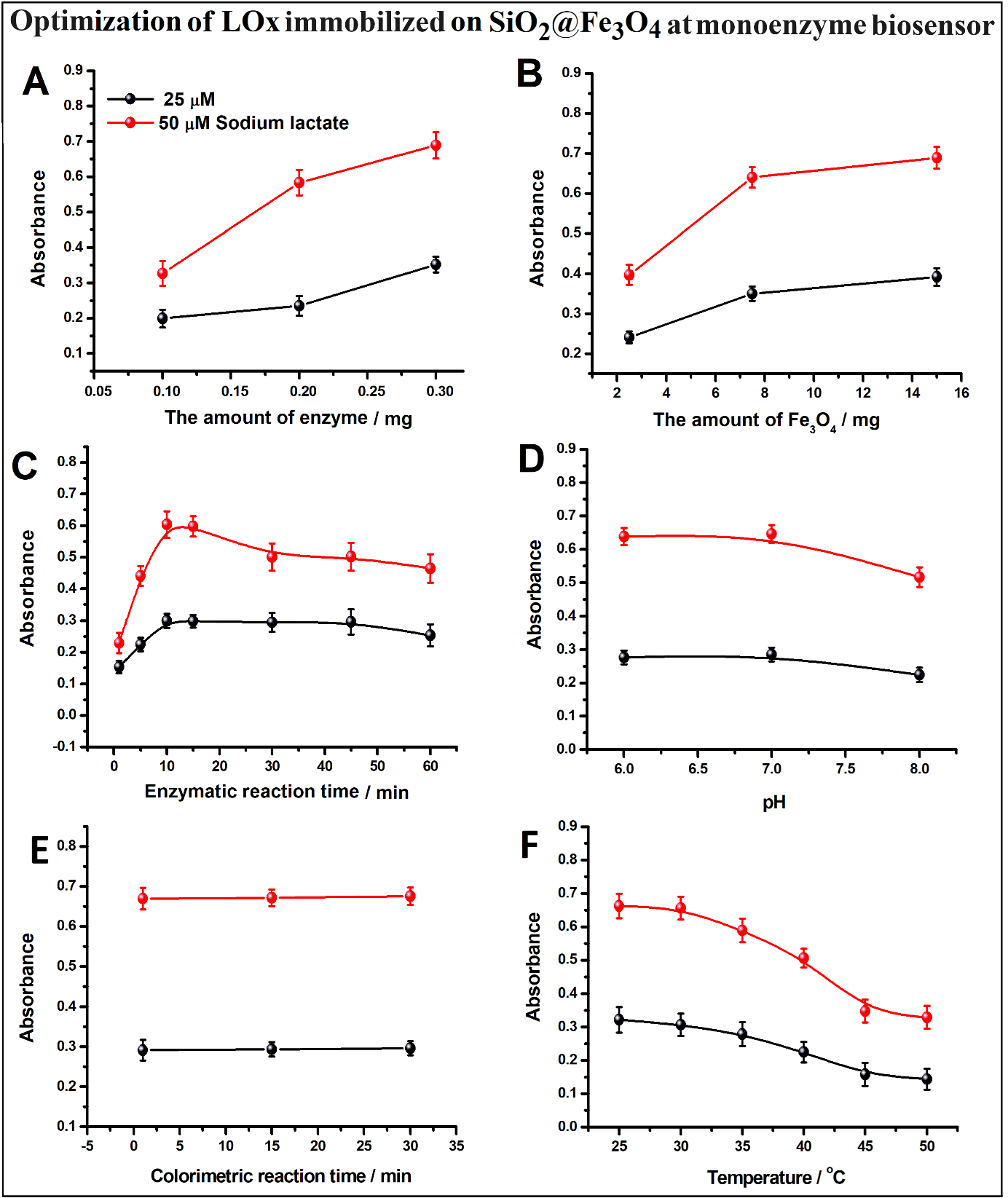
**

**Fig. S9.** Optimization curves of **A)** enzyme amount of immobilized LOx on 15 mg SiO_2_@Fe_3_O_4_, **B)** amount of Fe_3_O_4_ using 0.3 mg LOx, and following optimization curves of **C)** enzymatic reaction time, **D)** colorimetric reaction time, **E)** pH, and **F)** temperature using 15 mg monoenzyme immobilized Fe_3_O_4_ NPs (LOx (0.3 mg)@SiO_2_@Fe_3_O_4_ NPs)

**References**

[1] Ayaz S, Üzer A, Dilgin Y, Apak R (2023) Fabrication of a novel optical glucose biosensor using copper(II) neocuproine as a chromogenic oxidant and glucose dehydrogenase-immobilized magnetite nanoparticles. ACS Omega 8:47163–47172. <https://doi.org/10.1021/acsomega.3c07181>

[2] Malik M, Chaudhary R, Pundir CS (2019) Construction of an amperometric pyruvate biosensor based on enzyme bound to a nanocomposite and its comparison with enzyme nanoparticles bound to electrode. Int. J. Appl. Sci. Biotechnol. 7(2): 195-206 <https://doi.org/10.3126/ijasbt.v7i2.24445>

[3] Dagar K, Pundir CS (2016) Covalent immobilization of lactate oxidase onto zirconia coated silica

nanoparticles/chitosan hybrid film for amperometric determination of lactate, Biochem Anal Biochem 5:4- 1000301 <https://doi.org/10.4172/2161-1009.1000301>
